# Supplementary material for: First Detection of Tetrodotoxin in Bivalves and Gastropods from the French Mainland Coasts
Source: Toxins (Basel). 2020 Sep 16;12(9):599. doi: 10.3390/toxins12090599 (PMC7551754; doi:10.3390/toxins12090599)
Supplement: Supplementary file 1 [file toxins-12-00599-s001.pdf]

Supplementary Materials: First Detection of Tetrodotoxin in Bivalves and Gastropods from the French Mainland Coasts

Vincent Hort, Nathalie Arnich, Thierry Guérin, Gwenaëlle Lavison-Bompard and Marina Nicolas

- Weigh 5.0 ± 0.1 g of homogenized shellfish tissue

- Add 5 mL of 1% acetic acid to the test portion
- Shake for 90 s on a vortex mixer
- Place the sealed tube containing the sample a boiling water bath for 5 min
- Place the sealed tube containing the sample in a cold water bath for 5 min
- Shake for 90 s on a vortex mixer
- Centrifuge 10 min at 9000 g
- Transfer the supernatant in a 50 mL centrifuge tube

- Add 10 mL of 1% acetic acid to the sample
- Shake for 90 s on a vortex mixer
- Centrifuge 10 min at 9000 g
- Transfer the supernatant in a 50 mL centrifuge tube

- Adjust the volume to 20 mL with 1% acetic acid
- Centrifuge 5 min at 14 500 g

- Dilute the extract (1:10) with water/acetonitrile/acetic acid 88.9:11.1:0.167 (v/v/v)
- Filter the diluted extract through nylon syringe filter (0.22 µm)

Figure S1. Double extraction of tetrodotoxins (TTXs) from homogenized shellfish tissues.

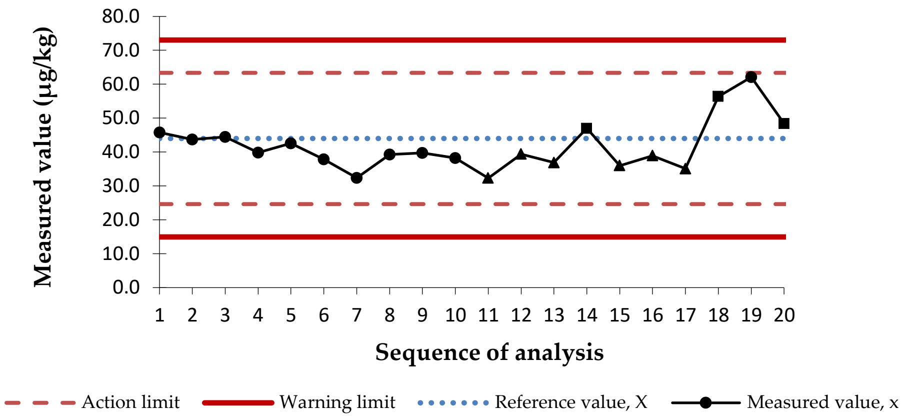

Figure S2. Control chart of blank matrices spiked at 44 µg TTX/kg and used as internal quality control (Round mark: mussel sample; triangle mark: whelk sample; square mark: oyster sample).
